# Evaluation of patients admitted with musculoskeletal tuberculosis: sixteen years’ experience from a single center in Turkey

**DOI:** 10.1186/s12891-021-04426-y

**Published:** 2021-06-14

**Authors:** Mikail Ozdemir, H. Gulnihal Ozdemir

**Affiliations:** 1 Osmaniye Tuberculosis Dispensary Public Health Department, Kazım Karabekir Street Musa Şahin Boulevard No:74/513 Center, 80010 Osmaniye, Turkey; 2 Osmaniye State Hospital Pathology Department, Akyar Central Location Hospital Street Center, 80010 Osmaniye, Turkey

**Keywords:** Bursitis, Extrapulmonary, Pott's spine, Tuberculosis dispensary

## Abstract

**Background:**

The aim of our study was to describe musculoskeletal system tuberculosis (TB) as a single-center experience.

**Methods:**

This is a retrospective observational study conducted at a TB Dispensary in the east Mediterranean part of Turkey between 2004 and 2020. The clinical and demographic characteristics including age, gender, involvement location and duration of illness, presenting complaint, local examination findings, treatment outcome were retrieved and analyzed from the case files. Statistical analyses were performed using SPSS Statistics version 17.0 (IBM). The normality of data analysed by using Kolmogorov-Smirnov. The descriptive statistics were reported as mean ± standard deviation, medians, and ranges (min-max).

**Results:**

Overall, 31 patients (3.2 % of all TB cases) with a mean age of 44.2 ± 16.7 years had musculoskeletal tuberculosis. The mean duration of treatment was 12.9 ± 5.5 months. Of the 31 patients, six (19.4 %) had concomitant pulmonary TB. One of the patients was in the pediatrics age group, and two of them were in the geriatric group. The most affected area was the vertebra. The most common complaint of the patients was back pain and seen in 22 patients (70.9 %).

**Conclusions:**

The physicians should be suspicious about the diagnosis of musculoskeletal TB disease. If the diagnosis and treatment are delayed, spinal damage and other consequences might be incurable.

## Introduction

Tuberculosis (TB) is a chronic infectious disease mainly caused by *Mycobacterium tuberculosis*. TB is a public health problem, especially in underdeveloped or developing countries, and remains a major cause of morbidity and mortality [1]. As reported by World Health Organization (WHO), the estimated global incidence of TB cases was 10.0 million in 2018 [2]. In Turkey, as a developing country, the TB incidence (the number of new TB cases per 100,000 populations per year) decreased annually, from 29.8 to 2005 to 14.4 in 2018 [3, 4].

More than 80 % of TB cases are pulmonary TB. However, several manifestations of extra-pulmonary TB have been reported, including musculoskeletal system TB [5]. Although pulmonary TB has been extensively covered in the literature [6–8], publications on musculoskeletal TB are relatively limited, and have been reported mostly in case reports [9–11]. In addition, the diagnosis of musculoskeletal system TB can be challenging, particularly for the physicians who are not familiar, because TB can present in atypical locations and mimic tumoral lesions [12]. Therefore, a biopsy should be taken to confirm the diagnosis.

Histopathologically, a granulomatous inflammatory reaction that occurs against mycobacterial species is composed of epithelioid histiocytes, giant cells, lymphocytes, and some of them have centrally located caseous necrosis. The granuloma can not only occur against TB disease, but also against fungi as an infectious agent, sarcoidosis as an immunological reaction, or against a foreign body. Histochemically, *Mycobacterium tuberculosis* bacilli can be demonstrated inside the granuloma, mainly in necrotic areas with Erlich-Ziehl-Neelsen (EZN) stain to confirm the diagnosis. Also, the diagnosis can be made by culture and amplification of *Mycobacterium tuberculosis* DNA using the polymerase chain reaction (PCR) method. The diagnosis of musculoskeletal TB is usually tricky and could be delayed. A positive skin tuberculin test or abnormal chest radiograph will support the diagnosis though it is not excluded by negative results [13].

Respecting immunosuppression, the incidence of musculoskeletal TB and all other extra-pulmonary TB infections depend on the grade of weakening cellular immunity. People with human immunodeficiency virus/acquired immunodeficiency syndrome (HIV/AIDS) and latent TB are also far more likely to progress active disease, with a nearly 10 % risk of developing active disease each year, in comparison with a lifelong reactivation risk of about 5 % in an HIV/AIDS-negative population. As impairment of the immune system progresses, patients living with HIV/AIDS are more likely to develop extra-pulmonary TB, just like musculoskeletal TB [14].

Keeping in mind the increased prevalence of TB in recent years [15], we deem it important to review musculoskeletal involvement of TB. Accordingly, the objective of this study was to elaborate musculoskeletal system TB as a single-center experience.

## Materials and methods

This retrospective study was conducted as a descriptive single-center experience. Patients who were followed up in Osmaniye Tuberculosis Dispensary between 2004 and 2020 were screened from the dispensary records. This dispensary is a governmental institution located in the south of the country with a population of approximately 350,000 (~ 90 % urban and ~ 10 % rural) that provides free service (including medicines) to all patients. In addition, all TB patients living in this area have to apply to this dispensary to provide directly observed therapy since it is the only TB center in the city. Among them, cases with musculoskeletal system involvement were noted. All patients were diagnosed with tuberculosis according to the histopathologic examinations by a pathologist. The biopsy samples were evaluated grossly and processed using the routine histopathologic technique (fixation and paraffin embedding) and then stained with Haematoxylin-Eosin.

Definite diagnosis of musculoskeletal TB was made when either (I) joint aspiration or tissue biopsy/fine-needle aspiration cytology (FNAC) revealed acid-fast bacilli (AFB) detection by smear examination, (II) culture of the synovial fluid (or tissue aspirated) grew *Mycobacterium tuberculosis*, or (III) histopathology/FNAC revealed granulomatous lesions with or without caseation with AFB positivity. Probable TB was considered if there was no explicit evidence of AFB or granuloma, but clinical, radiological, and serological evidence suggested TB and the patient responded to empirical antitubercular therapy. Other tests include tuberculin skin sensitivity (PPD) test and TB interferon γ-assays. Radiology included X-ray, magnetic resonance imaging (MRI), or computed tomography (CT) of the organ involved. CT-guided biopsy or fine-needle aspiration and arthroscopic biopsies were also performed in some cases. The clinical and demographic characteristics, including age, gender, involvement location and duration of illness, presenting complaint, local examination findings, treatment outcome were retrieved and analyzed from the case files. TB is a notifiable disease, and data are recorded prospectively by our specialist TB nurses. Patients with missing data in terms of diagnosis, clinical follow-up, or treatment, and patients transferred to another tuberculosis center were excluded.

Typical treatment of all cases included the use of a four-drug regimen: isoniazid, rifampicin, ethambutol, and pyrazinamide for the initiative phase of two months, followed by 4–10 months of continuation phase with isoniazid and rifampicin. If the case was a recurrence TB, streptomycin applied to the patients intravenously (IV) is added to the treatment regimen for first two months. The drug doses were adjusted according to the weight of the patients. Drug susceptibility testing for the first-line drugs was conducted for patients who did not respond to treatment, and regimens were changed based on the susceptibility results or strong clinical suspicion of unresponsiveness.

Statistical analyses were performed using SPSS Statistics version 17.0 (IBM). The normality of data analyzed by using Kolmogorov-Smirnov. The descriptive statistics were reported as mean ± standard deviation, medians, and ranges (min-max).

The current study protocol was approved by the Local Ethics Committee of Adana City Training and Research Hospital (decision number: 321/2018). This study was exempted from informed consent form by this ethics committee, since it was a retrospective study. All authors confirmed that all methods were performed in accordance with the relevant guidelines and regulations.

## Results

A total of 949 patients were followed up due to tuberculosis in our center between 2004 and 2020. Overall, 31 (3.2 % of all TB cases) patients (14 males, 17 females) with a mean age of 44.2 ± 16.7 years (ranges: 3 to 75 years) had musculoskeletal tuberculosis. Demographic features are summarized in Table 1.

**Table 1:** Demographic Features

Variables	Results	
**Age** (years)^a^	44.2± 16.7	49 (3-75)
**Gender** n, (%)		
- Male	14 (45.2)	
- Female	17 (54.8)	
**Age group** (years) n, (%)		
- Pediatric (<18)	1 (3.2)	
- Adult (18-64)	28 (90.3)	
- Geriatric (≥ 65)	2 (6.5)	
**Race** n, (%)		
- Turkish	29 (93.5)	
- Afghanistan	1 (3.2)	
- Syria	1 (3.2)	
**Occupation** n, (%)		
- Housewife	12 (38.7)	
- Butcher	4 (12.9)	
- Student	4 (12.9)	
- Retired	4 (12.9)	
- Worker	2 (6.5)	
- Farmer	2 (6.5)	
- Cooker	1 (3.2)	
- Craftsman	1 (3.2)	
- Cooker	1 (3.2)	
**Family history of TB** n, (%)	6 (19.4)	
**Concomitant Pulmonary TB** n, (%)	6 (19.4)	

*TB* Tuberculosis

^a^mean ± S.D and median (minimum-maximum)

**Fig. 1:**
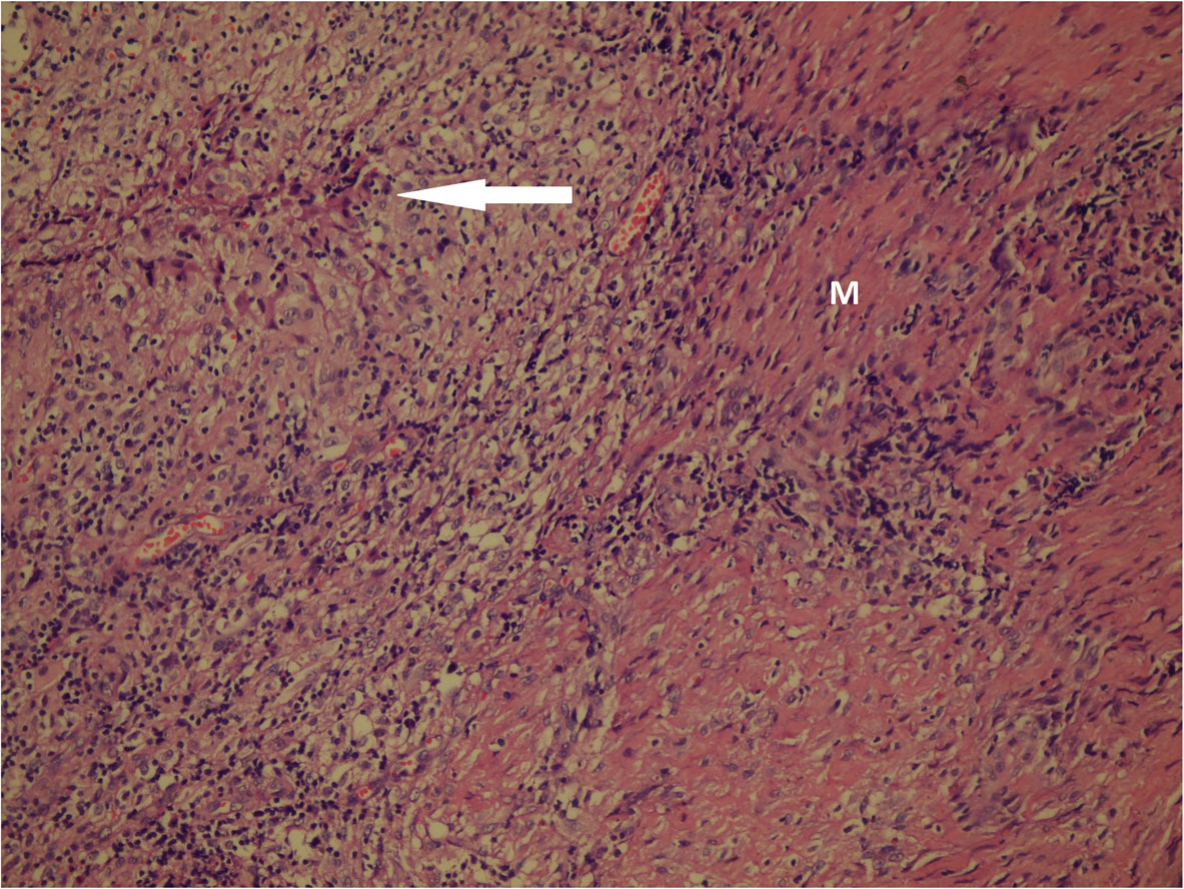
Granulomatosis inflammatory reaction against *M. tuberculosis* with caseous necrosis (white arrow) seen in muscle tissue (M) (×200, H&E)

**Fig. 2:**
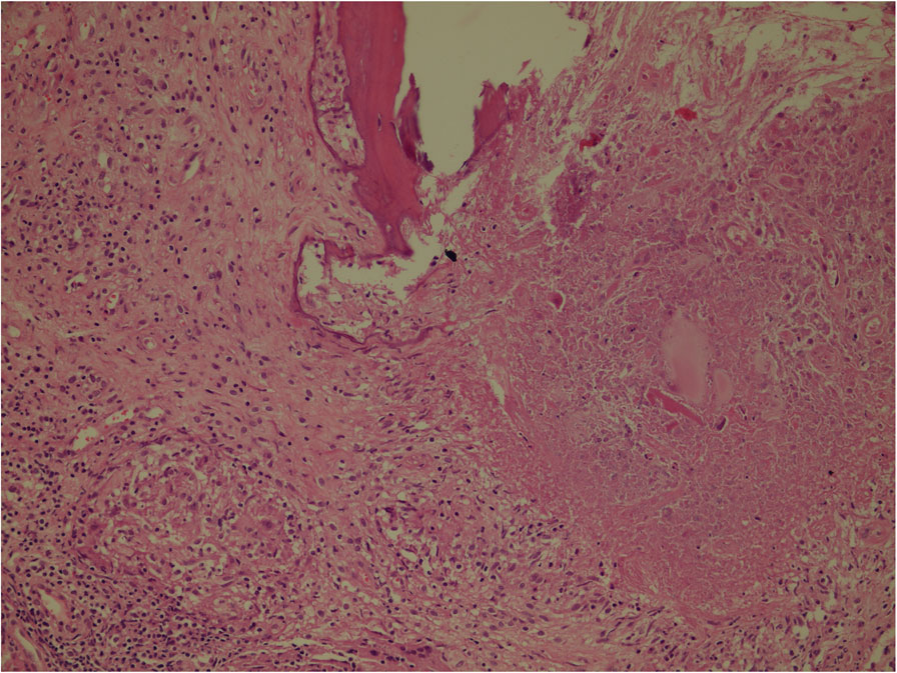
Granulomatous inflammatory reaction involving caseified necrosis that destroys bone tissue (x200, H&E)

Clinical presentations of the cases are given in Table 2. Back pain (*n* = 22, 70.9 %) and shoulder pain (*n* = 2, 6.5 %) were the most seen symptoms, and thoracic or lumbar vertebra was the most involved location (74.2 %). The example images of a shoulder and thoracic vertebra TB are also shown in Figs. 1 and 2, respectively. Most of the cases (58.1 %) were diagnosed in tertiary hospitals. The mean duration of treatment was 12.9 ± 5.5 months (range, 1.3 week to 27.7 months). Two of the patients had a recurrent illness and were treated with the HRZE + S (isoniazid, rifampicin, ethambutol, and pyrazinamide + IV streptomycin) regimen (Table 2).

**Table 2:** Clinical Presentation

**Variable**	**n**	**%**
**Symptoms**		
- Back pain	22	70.9
- Mass on back and neck	1	3.2
- Knee pain	1	3.2
- Wrist swelling	1	3.2
- Chest pain	1	3.2
- Hand pain	1	3.2
- Shoulder pain	2	6.5
- Abdominal/hip pain	1	3.2
**Location**		
- Vertebra	23	74.2
- Hand	2	6.5
- Knee joint	1	3.2
- Costa	1	3.2
- Shoulder	1	3.2
- Patella	1	3.2
- Psoas muscle	2	6.5
**Vertebra Location**		
- Thoracic	10	32.3
- Lumbar	12	38.7
- Sacrococcygeal	1	3.2
**Center of Diagnosis**		
- Secondary	13	41.9
- Tertiary	18	58.1
**Treatment duration** (months)^a^	12.9± 5.5 & 12.0 (1.3-27.7)	
**Outcome**		
- **Treatment completed**	26	83.8
- **Treatment is going on**	3	9.6
- **Exitus**	1	3.2
- **Leaved treatment**	1	3.2
**Primer TB**	29	93.5
**Recurrence TB**	2	6.5
**Treatment Regimen**		
- **HRZE**	29	93.5
- **HRZE + S**	2	6.5

*TB* Tuberculosis, *HRZE* isoniazid + rifampicin + ethambutol + pyrazinamide, *HRZE + S* isoniazid + rifampicin + ethambutol + pyrazinamide + streptomycin

^a^mean ± S.D & median (percentile 25-percentile 75)

### Bursit case

**Fig. 3:**
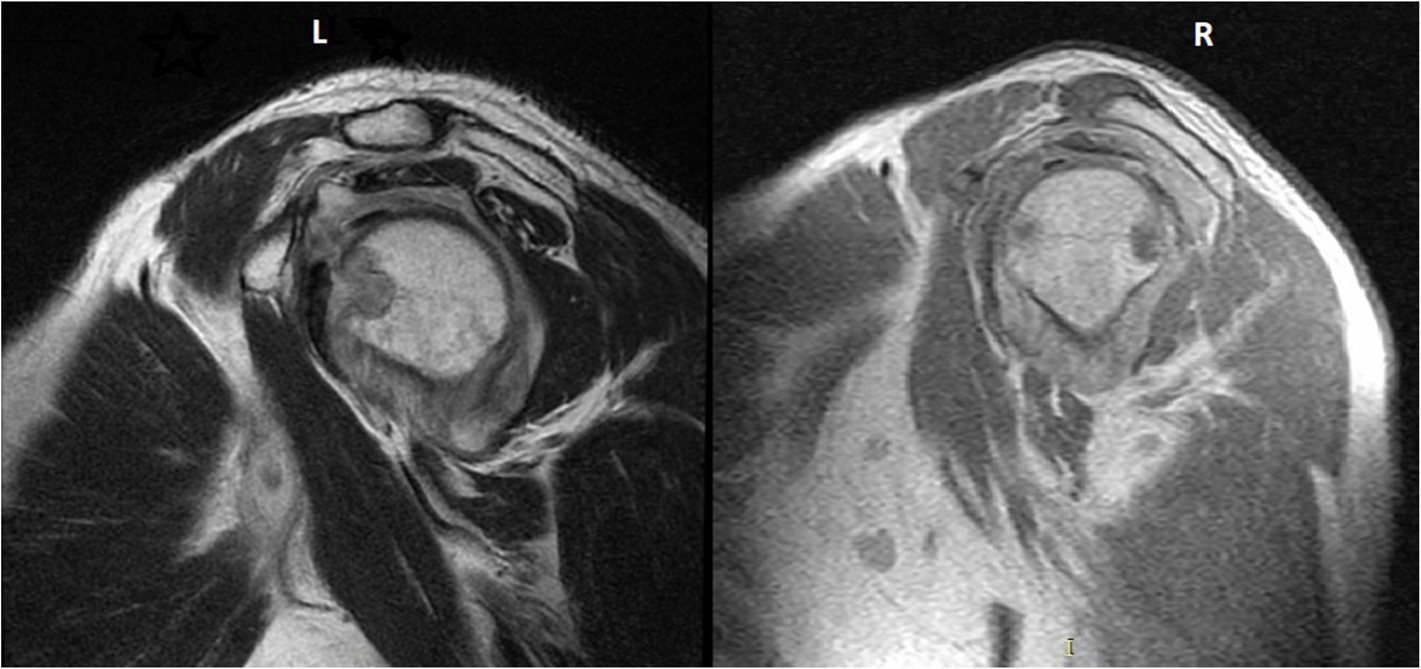
Preoperative (left) and postoperative (right) Magnetic Rezonans Imaging (sagittal plane T1) of bursitis case

The example images are from a 49-year-old man who presented with fatigue, left shoulder pain, and restriction in shoulder movements (Fig. 3). Laboratory investigations yielded increased levels of C-reactive protein and erythrocyte sedimentation rate. MRI showed joint effusion, synovial hypertrophy, bursitis, rice bodies, and cortical erosions. The pathological examination was consistent with lymphohistiocytic inflammatory response, including giant cells and caseified necrosis in adipose and connective tissue (i.e., granulomatous inflammatory response). The patient was received a 12 month-treatment (2 months HRZE + 10 months HR). After 12 months of treatment, the patient’s treatment file was closed as ‘treatment completion’ after his complaints completely disappeared.

## Discussion

The majority of extra-pulmonary TB cases involve the pleura, musculoskeletal and lymphatic systems. Most cases verified to have a previous pulmonary TB origin [16]. TB bacilli spread over the pleural cavity, starting from a pulmonary infection focus on that occasion, immigrates through the blood vessels or lymphatics to other organs developing extrapulmonary TB.

The distribution of age and site of musculoskeletal TB has been previously studied. Overall, of all TB cases, 1-4 % showed musculoskeletal involvement. In a retrospective and observational study conducted in a tertiary area with the highest prevalence of TB worldwide, three peaks in the first, third, and sixth decade of life were found [17]. However, some reports highlighted a bimodal age distribution [18]. In a nine-year single-center experience, the rate of musculoskeletal TB was found as 4 % [19]. In our study, the rate of musculoskeletal system TB was 3.2 %, which was compatible with the previous reports. On the other hand, the mean age was 44 years, and 90 % of all cases were adult patients. We did not find an age peak in childhood or in the advanced age group. Musculoskeletal TB is distributed almost equally among males and females in our sample group.

In the literature, most of the TB cases present with spine involvement. More than 10 % of patients with extra-pulmonary TB have skeletal involvement [20]. The most common form of skeletal TB is the Pott’s disease, comprising approximately half of musculoskeletal TB cases, and followed by TB arthritis and extra-spinal TB osteomyelitis [21]. In the retrospective study done by Held et al. [17], 78 % of the cases had spine TB while the remaining 21.6 % had extra-spinal diseases, comprising hip, knee, foot/ankle, shoulder, elbow, wrist, and others. The thoracic region of the vertebral column is commonly involved [20]. Likewise, in our study, the prevalent anatomically affected location was the spine (23 of 31 patients; 75 %) as well as hands, knee joint, costa, shoulder, patella, and psoas muscle. Only two cases in this study were involved with psoas muscle. These two cases had both concomitant pulmonary TB. The psoas muscle is a retroperitoneal muscle that originates from the lateral borders of T12 to L5 vertebrae and ends as a tendon that inserts into the lesser trochanter. The primary TB of iliopsoas compartment abscess with occult cause rarely encountered in the clinical practice and is generally idiopathic.

The joints’ TB infection comes after hematogenous spread or direct invasion from neighboring tissue of TB osteomyelitis. Mostly monoarticular joints are involved such as the knee and hip. Oligoarticular/polyarticular patterns are very rare, ranging from 5 to 15 % of cases, sometimes with small joint involvement, and ordinary in immunosuppressed patients [22]. A patient in our series (male, 38-year-old, butcher), TB was detected in the biopsy performed a long time after the local infectious swelling developed with a knife sticking in the left hand at the workplace.

The delays in the diagnosis of musculoskeletal TB have been sufficiently presented [13]. This may be due to the patients’ uncertain histories, perhaps complicated by inaccurate stories of irrelevant trauma, and lack of presence of a concomitant pulmonary involvement. In the current study, complaints of 70.9 % of cases have been started as back pain. Therefore, these patients have been investigated in neurosurgery, orthopedic and traumatology, or algology clinics for a long time. The patients usually presented with nonspecific symptoms such as back pain, mass on back/neck, knee pain, wrist swelling, chest pain, shoulder pain, hand pain, or abdominal pain.

There were some limitations to our work. Primarily, because of the retrospective nature of our study, it was not possible to obtain detailed information of every patient. Furthermore, because the patient registry systems of dispensaries and hospitals are not integrated, monitoring and clinical information, such as misdiagnosis or delayed diagnosis for other reasons, that are not recorded in the ledger could not be accessed. In Turkey, all patients suspected with TB, whether they are pediatrics or adults, are directed to dispensaries to confirm their diagnosis and to give their treatment. However, dispensaries do not have a chance to diagnose possible overlooked cases unless clinicians from other branches express suspicion of TB. Even if all musculoskeletal TB patients living in this area have to apply to this dispensary as it is the only TB center in the city, the present results should not be generalized to the entire Turkey.

## Conclusions

To sum up, although it is a very rare disease, surgeons and clinicians should be aware of the diagnosis of musculoskeletal TB infection especially the presence of pain not relieved despite the physical therapy and B symptoms such as fever and weight loss. Further, in these cases, confused with cancer clinically and radiologically, it would be appropriate to take a biopsy to make the diagnosis at an earlier stage and exclude the cancer. In addition, cases in which granulomatous inflammatory reaction is reported should be referred to the dispensary immediately. On the assumption that the diagnosis and treatment is delayed, spinal damage in addition to the bones and joints of the patients may be an inevitable consequence. Consequently, musculoskeletal TB needs a complex approach and cooperation.

## Data Availability

The datasets used in this study are available from the corresponding author on reasonable request.

## Abbreviations

AFB: Acid-fast bacilli
CT: Computed tomography
EZN: Erlich-Ziehl-Neelsen
FNAC: Fine-needle aspiration cytology
HIV/AIDS: Human immunodeficiency virus/acquired immunodeficiency syndrome
HRZE + S: Isoniazid, rifampicin, ethambutol, and pyrazinamide + streptomycin
IV: Intravenously
MRI: Magnetic resonance imaging
PCR: Polymerase chain reaction
PPD: Tuberculin skin sensitivity
TB: Tuberculosis
WHO: World Health Organization
